# Macular hole edge morphology predicts restoration of postoperative retinal microstructure and functional outcome

**DOI:** 10.1186/s12886-020-01541-7

**Published:** 2020-07-11

**Authors:** Jiwei Tao, Huan Chen, Lin Zhu, Deming Pan, Jia Fang, Yiqi Chen, Jianbo Mao, Lijun Shen

**Affiliations:** Department of Retina Center, Affiliated Eye Hospital of Wenzhou Medical University, Hangzhou, Zhejiang Province China

**Keywords:** Intraoperative optical coherence tomography, Internal limiting membrane peeling, Macular hole, Retina microstructure, Visual outcomes

## Abstract

**Background:**

To investigate the ability of intraoperative optical coherence tomography (iOCT) during macular hole (MH) surgery to image different hole edge configurations and predict the restoration of retinal microstructure and visual outcomes.

**Methods:**

This retrospective case series study included 53 MH patients. One eye each was assessed with iOCT during vitrectomy after internal limiting membrane (ILM) peeling. The MHs were categorized into three groups according to the morphology of the hole edge. The Hole-Door group had vertical pillars of tissue that projected into the vitreous cavity after ILM peeling. The Foveal Flap group had a preoperative foveal flap that adhered to the hole edge after ILM peeling, and the Negative group had neither a hole-door nor a foveal flap. At 6 months after surgery, the retinal microstructure restoration and visual outcomes were compared among the groups.

**Results:**

All eyes had MH closure, and the postoperative best corrected visual acuity (BCVA) was significantly improved compared with the preoperative BCVA (*P* < 0.001). The Hole-Door group (*n* = 15) and Foveal Flap group (*n* = 14) had significantly better final visual acuity and postoperative restoration of the external limiting membrane (ELM) than the Negative group (*n* = 24) (*P* = 0.002, *P* = 0.012). For the group in which the MH diameter (MHD) was ≤400 μm (*n* = 25), there were no significant differences in ELM restoration, ellipsoid zone (EZ) restoration, or BCVA among the three groups (*P* = 0.516, *P* = 0.179, and *P* = 0.179 respectively). For the MHD > 400-μm group (*n* = 28, the Hole-Door group and Foveal Flap group had significantly better final visual acuity and restoration of ELM than the Negative group (*P* = 0.013, *P* = 0.005).

**Conclusions:**

The novel use of iOCT during MH surgery confirmed the presence of hole edges configured as door-holes, foveal flaps, or neither. The data acquired by iOCT can provide useful predictive information for postoperative restoration of the retinal microstructure and visual outcome of MH, especially large ones.

## Background

Full-thickness macular holes (MHs) can lead to decreased central vision and metamorphopsia [1–4]. Currently, the standard surgical procedure includes pars plana vitrectomy (PPV), internal limiting membrane (ILM) peeling, intraocular gas tamponade, and postoperative positioning to ensure satisfactory anatomic outcomes with successful MH closure rates of 90% [1–4]. Recent studies, using spectral domain optical coherence tomography (SD-OCT) to image retinal microstructure, reported favorable visual outcomes after MH surgery, especially regarding restoration of the external limiting membrane (ELM) and the ellipsoid zone (EZ) [5, 6]. Several studies have indicated that the recovery of the ELM might be the most critical factor for visual function improvement in the early postoperative period [7, 8].

Researchers have used OCT measurements in attempts to evaluate prognostic factors in MH surgery, including minimum hole diameter (MHD), base hole diameter, hole form factor, macular hole index, and tractional hole index [9–12]. However, the results have been variable due to the poor reproducibility of the OCT measurements. Recent studies have proved the feasibility and usefulness of intraoperative OCT (iOCT) in vitreoretinal surgery [13–15]. iOCT was said to have added valuable information related to surgical anatomic features, and it directly impacted the surgical procedure. Other recent studies have shown the feasibility of real time iOCT in PPV surgery [14, 15]. The DISCOVER study reported that the majority of surgeons preferred viewing static images rather than real-time images. Therefore, non-real–time iOCT still has important clinical value in vitreoretinal surgery [13]. Several authors have recently shown iOCT to be useful in patients undergoing membrane peeling in MH surgery [16, 17]. Moreover, alterations in MH geometry on iOCT have been visualized that may have important implications for postoperative care and positioning [16, 17]. However, the relationship of these intraoperative changes with the successful MH closure rate and anatomic normalization have not been well analyzed.

In this study, we used iOCT after ILM peeling during vitrectomy for MH to describe morphological changes at the edges of the MHs. With iOCT imaging, we identified three types of MHs based on the morphology of the hole edge after ILM peeling. We then analyzed the postsurgical association of the hole edge types with the restoration of retinal microstructure and postoperative visual outcomes.

## Methods

### Study design

This was a retrospective study of consecutive patients undergoing 23-gauge PPV for MH by a single surgeon (L.J-S) at the Department of Retina Center, Affiliated Eye Hospital of Wenzhou Medical University, Hangzhou, Zhejiang Province, China, from July 2015 to July 2018. All patients gave written informed consent prior to the surgery. All procedures were approved by the institutional review board of the Eye Hospital of Wenzhou Medical University and adhered to the Declarations of Helsinki.

### Patient selection

All patients with a MH who underwent 23-gauge PPV were included in the study. MH was define as a full-thickness retinal defect in the foveal neurosensory retina as visualized by SD-OCT. Exclusion criteria included previous vitreoretinal surgery, history of penetrating trauma, degenerative myopia, final follow-up period less than 6 months, and eyes that underwent PPV for MH without iOCT or by other techniques, e.g., inverted ILM flap technique, during the study period.

**Fig. 1:**
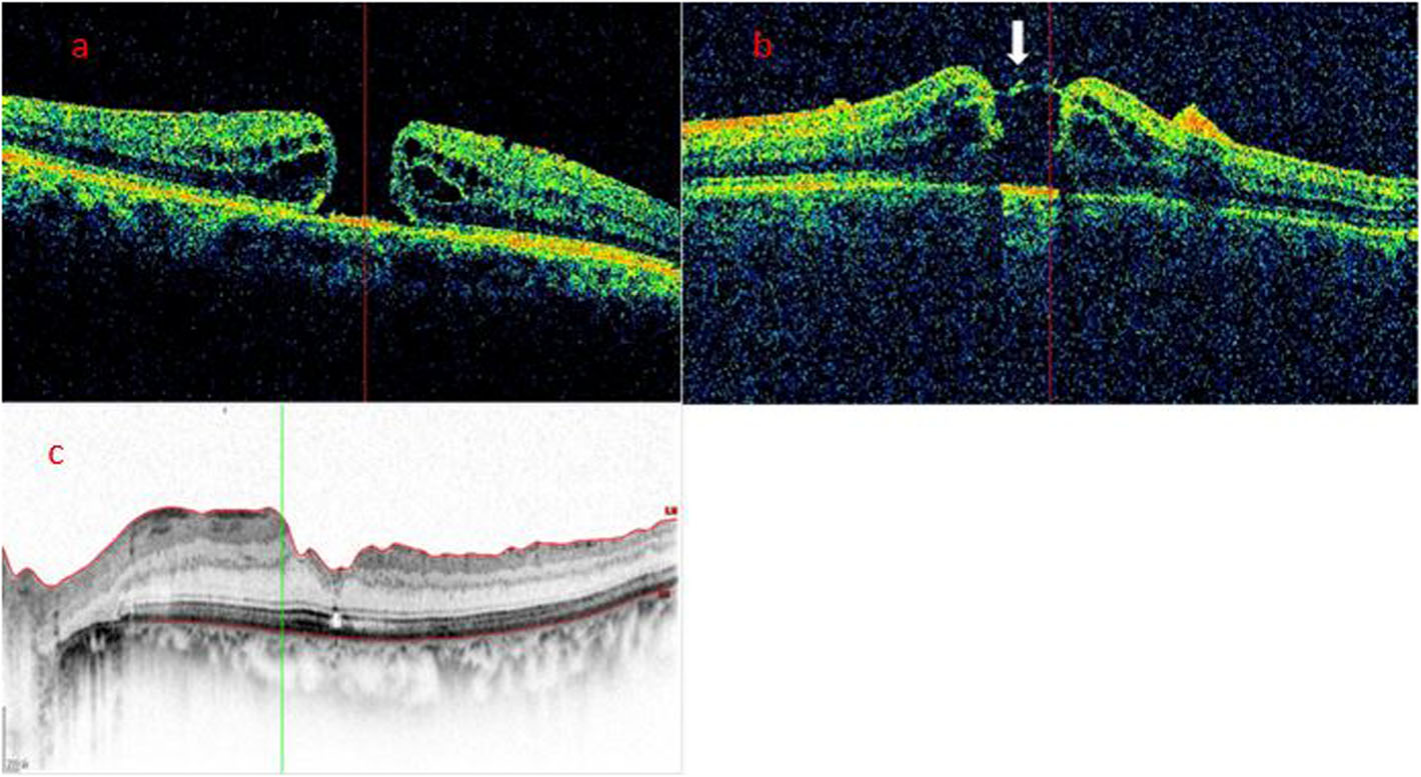
Case 1: A representative hole-door case. **a** The preoperative SD-OCT image showed a MH without a foveal flap in a 60-year-old woman. **b** iOCT showed vertical pillars of tissue at the edges of the hole projecting into the vitreous cavity (arrow) after ILM peeling. **c** Postoperatively at 6 months, SD-OCT showed hole closure with full recovery of the ELM and EZ. The BCVA was 0.4

**Fig. 2:**
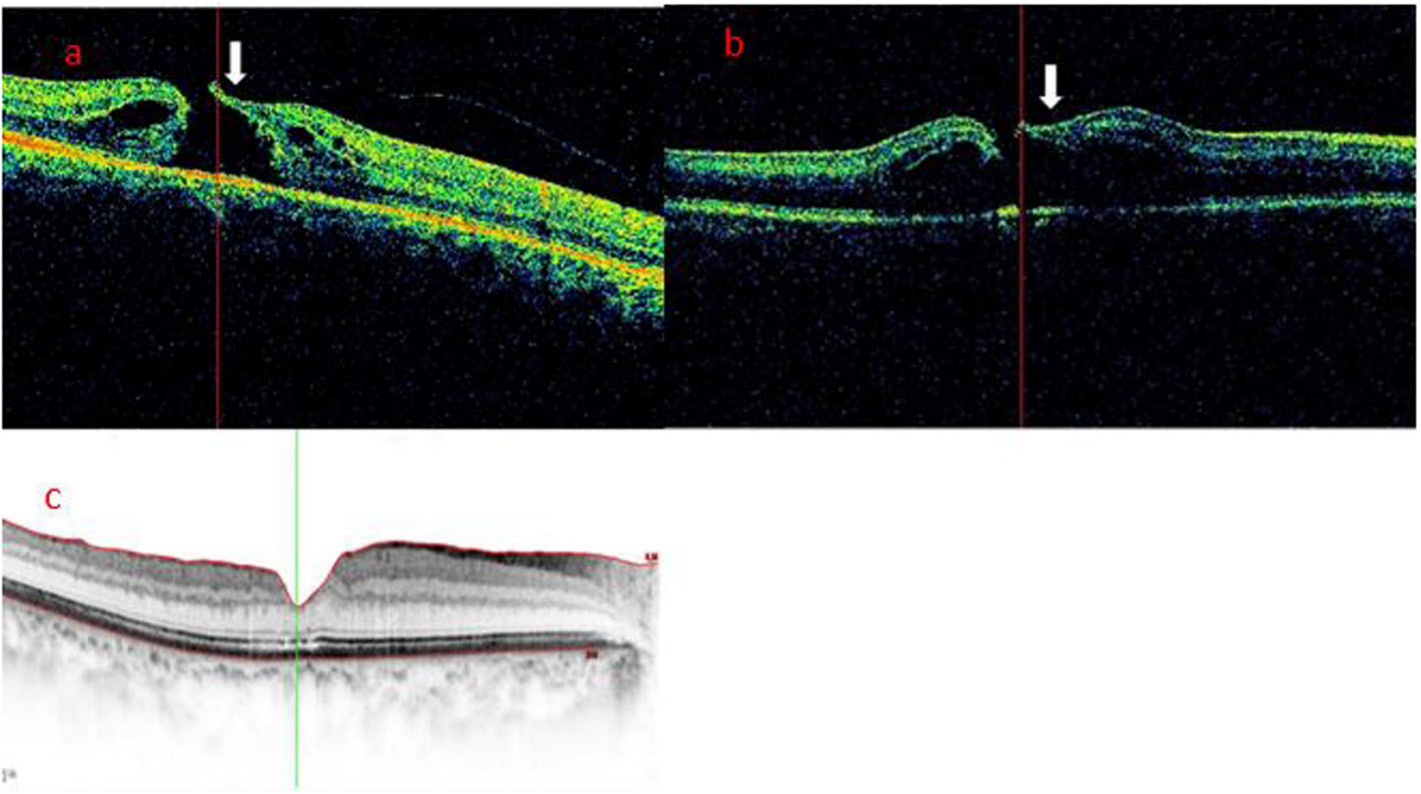
Case 2: A representative foveal flap case. **a** The MH foveal flap was evident in the preoperative SD-OCT images (arrow) of a 63-year-old woman. **b** iOCT showed that the foveal flap (arrow) was preserved after ILM peeling. **c** Postoperatively at 6 months, SD-OCT showed hole closure with recovery of the ELM and EZ. The BCVA was 0.6

**Fig. 3:**
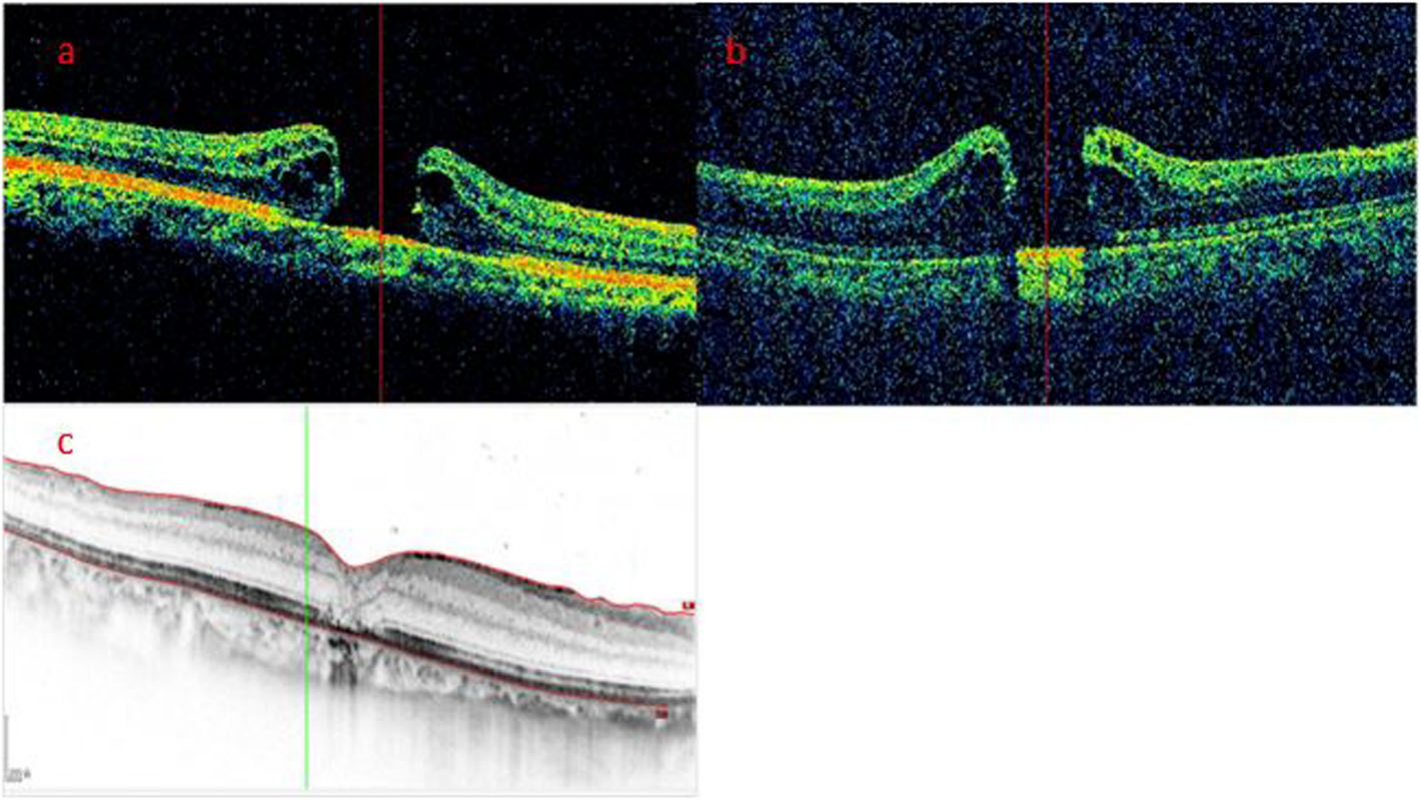
Case 3: A representative negative case. **a** The preoperative SD-OCT image showed a MH without a foveal flap in a 67-year-old woman. **b** iOCT showed neither foveal flap nor vertical pillars of tissue at the edges of the hole after ILM peeling. **c** Postoperatively at 6 months, SD-OCT showed hole closure without restoration of the ONL. The bridging tissue was hyperreflective. The BCVA was 0.15

According to the morphological characteristics of the hole edge as imaged by iOCT after ILM peeling, all patients were divided into three groups. In the Hole-Door group, iOCT revealed vertical pillars of tissue that projected into the vitreous cavity from the edges of the hole (Fig. 1). In the Foveal Flap group, iOCT imaged a preoperative foveal flap that adhered to the hole edge after ILM peeling (Fig. 2). In the Negative group, iOCT imaged neither hole-door nor foveal flap features (Fig. 3).

### Surgical technique

Each surgery was performed under retrobulbar anesthesia in patients receiving 23-gauge PPV. After core vitrectomy, posterior vitreous detachment was induced using the suction power of a 23-gauge vitrectomy cutter in the optic disc area. The posterior hyaloid membrane was cut except for the macular area. After staining the posterior pole with indocyanine green (0.025 mg/ml) for 5 s, the ILM was grasped with ILM forceps and peeled off for approximately two-to-three disc diameters around the MH. Air-fluid exchange was followed by C3F8 endotamponade.

All eyes underwent examination for best corrected visual acuity (BCVA) by Snellen chart. BCVA was expressed as the logarithm of the minimum angle of resolution (logMAR). The health of each anterior segment and fundus were also assessed by slit-lamp biomicroscopy and fundus photography respectively. All preoperative and postoperative OCTs were done using a commercially available SD-OCT device (Spectralis HRA OCT; Heidelberg Engineering, Heidelberg, Germany). Diameters of MH were defined as the shortest distances between the edges of the broken ends of the neuroepithelia on the largest cross-section and were quantified based on a horizontal scan through the center of the hole. iOCT images were obtained with the Optovue iVue OCT System (Optovue, Inc., Fremont, CA, USA). The scanning speed was 26,000 times/min, the vertical resolution was 5 μm, the horizontal resolution was 11.4 μm, and the wavelength was 830 nm. Images acquired before and after ILM peeling were analyzed for qualitative changes. The primary outcome measures were anatomic success and restoration of the photoreceptor layer of the MH as documented by SD-OCT. The functional outcome of surgery was evaluated by BCVA at the last follow-up.

The postoperative BCVA and anatomical morphology of photoreceptor layer observed on SD-OCT images obtained in the postoperative follow-up period were compared among the 3 MH groups. Moreover, the restoration of the photoreceptor layer was assessed via the reconstruction of the continuous back reflection line corresponding to the EZ and the ELM. Two independent observers (H.C, J.F) evaluated the images, with a consensus used to resolve disagreements. All data, were analyzed using the SPSS 22.0 statistical software (SPSS Inc., Chicago, IL, USA), and *P*-values ≤0.05 were considered to be statistically significant. One-way analysis of variance with post-hoc examination was used for the pre- and postoperative BCVA among the three groups throughout the study period. The Chi-squared test was applied to evaluate differences in macular hole closure and restoration of the ELM and EZ.

## Results

One eye each of 53 patients (36 females, 17 males) matched the study criteria and were included in the analysis. The mean age was 66.0 ± 6.5 years (range 47–78 y), and the preoperative BCVA for all subjects was 1.10 ± 0.77. The mean MHD was 420.19 ± 170.79 μm.

Based on the features of the hole edge as revealed by iOCT, the Hole-Door group included 15 eyes, the Foveal Flap group included 14 eyes, and the Negative group included 24 eyes (Table 1). At the baseline examination, there was no significant difference among the three groups regarding age (*P* = 0.151), sex (*P* = 0.252), axial length (*P* = 0.615), duration of MH (*P* = 0.735), mean preoperative BCVA (*P* = 0.287), or MHD (*P* = 0.268).

**Table 1:** Characteristics of the negative, hole-door, and foveal flap MH groups

	Groups			
	Hole-Door (***N*** = 15)	Foveal Flap (***N*** = 14)	Negative (***N*** = 24)	P*
Male/female, no	5/19	7/8	5/9	0.252
Age, (years)	62.3 ± 15.5	63.0 ± 7.3	68.1 ± 5.9	0.151
Axial length, (mm)	23.53 ± 0.94	23.33 ± 1.27	23.36 ± 0.77	0.615
Duration of MH, (months)	2.87 ± 1.51	3.00 ± 1.71	3.07 ± 1.92	0.735
Preoperative BCVA	1.34 ± 1.17	0.89 ± 0.44	1.06 ± 0.56	0.287
Preoperative MHD	391.47 ± 165.32	380.07 ± 166.61	463.35 ± 174.04	0.268
Macular hole closure	15 (100%)	14 (100%)	23 (95.8%)	0.717
ELM restoration	14 (93.3%)	13 (92.9%)	14 (58.3%)	0.012
EZ restoration	5 (33.3%)	7 (50%)	8 (33.3%)	0.587
Postoperative BCVA	0.24 ± 0.15	0.25 ± 0.15	0.68 ± 0.60	0.002

*MH* Macular hole, *BCVA* Best corrected visual acuity, *MHD* Minimum hole diameter, *ELM* External limiting membrane, *EZ* Ellipsoid zone; *, for age, axial length, duration of MH, preoperative BCVA, preoperative MHD, and postoperative BCVA, *P*-values were determined by ANOVA. For male/female ratio, MH closure, ELM restoration, and EZ restoration, *P*-values were determined by Chi-squared test

### MH closure, iOCT features, and visual acuity change after surgery

All of the eyes had MH closure after vitrectomy. Type 1 MH closure, in which there was complete closure and no bare retinal pigment epithelium, occurred in 52 eyes. One eye had a Type 2 MH closure in which the closure was incomplete, leaving some bare retinal pigment epithelium exposed. The postoperative BCVA for all subjects was 0.44 ± 0.46, which was significantly improved compared with the preoperative BCVA of 0.93 ± 0.47 (*P* < 0.001). Six months after surgery, the ELM was restored in 77.4% (41 of 53) of the patients. At the same time, the EZ was restored in 37.7% (20 of 53) of the patients.

### iOCT features and visual acuity change after surgery among the hole-door, Foveal flap, and negative groups

At 6 months after surgery, there was no significant difference in the percent of patients with MH closure among the three groups (Table 1). The postoperative visual acuity at that time was 0.24 ± 0.15 in the Hole-Door group, 0.25 ± 0.15 in the Foveal Flap group, and 0.68 ± 0.60 in the Negative group. The Hole-Door group and Foveal Flap group had significantly better final visual acuity than the Negative group (*P* = 0.002). The ELM was restored by 6 months after surgery in 93.3% (14 of 15) in the Hole-Door group, 92.9% (13 of 14) in the Foveal Flap group, and 58.3% (14 of 24) of the patients in the Negative group. Postoperative restoration of the EZ was present at 6 months in 33.3% (5 of 15) in the Hole-Door group, 50% (7 of 14) in the Foveal Flap group, and 33.3% (8 of 24) of the patients in the Negative group,. The Negative group had significantly poorer restoration of the ELM than the other two groups (*P* = 0.012, Table 1).

### Macular hole size, BCVA, and microstructural changes of the fovea after surgery

Subgroup analysis divided patients into groups in which the MHD was ≤400 μm (Table 2) or > 400 μm (Table 3). By 6 months after surgery, for the group in which the MHD was ≤400 μm, the ELMs were completely restored in all 3 MH groups (Table 2). At the same time, restoration of the EZ was achieved in 62.5% (5 of 8) in the Hole-Door group, 50% (4 of 8) in the Foveal Flap group, and 77.8% (7 of 9) of the patients in the Negative group. There were no significant differences in ELM restoration, EZ restoration, or BCVA among the three groups.

**Table 2:** Functional and anatomical outcomes in the MHD ≤400 mm group

	Groups			
	Hole-Door (***N*** = 8)	Foveal Flap (***N*** = 8)	Negative (***N*** = 9)	P
Preoperative BCVA	1.71 ± 1.53	0.90 ± 0.57	0.77 ± 0.28	0.11
Preoperative MHD, μm	285.75 ± 110.94	252.63 ± 72.19	289.56 ± 69.41	0.636
Macular hole closure	8 (100%)	8 (100%)	9 (100%)	/
ELM restoration	8 (100%)	8 (100%)	9 (100%)	/
EZ restoration	5 (62.5%)	4 (50%)	7 (77.8%)	0.516
Postoperative BCVA	0.16 ± 0.14	0.23 ± 0.18	0.30 ± 0.12	0.179

*MHD* Minimum hole diameter, *ELM* External limiting membrane, *EZ* Ellipsoid zone, *BCVA* Best corrected visual acuity; *, for preoperative BCVA, preoperative MHD, and postoperative BCVA, *P*-values were determined by ANOVA. For EZ restoration, *P*-value determined by Chi-squared test

**Table 3:** Functional and anatomical outcomes in the MHD > 400 mm group

	Groups			
	Hole-Door (***N*** = 7)	Foveal Flap (***N*** = 6)	Negative (***N*** = 15)	P
Preoperative BCVA	0.92 ± 0.26	0.89 ± 0.21	1.24 ± 0.62	0.205
Preoperative MHD, mm	512.29 ± 132.22	550.00 ± 65.11	575.07 ± 118.08	0.496
Macular hole closure	7 (100%)	6 (100%)	14 (93.3%)	0.75
ELM restoration	6 (85.7%)	6 (100%)	5 (33.3%)	0.005
EZ restoration	2 (28.6%)	2 (33.3%)	2 (13.3%)	0.569
Postoperative BCVA	0.34 ± 0.17	0.28 ± 0.10	0.93 ± 0.65	0.013

*MHD* Minimum hole diameter, *ELM* External limiting membrane, *EZ* Ellipsoid zone, *BCVA* Best corrected visual acuity; *, For preoperative BCVA, preoperative MHD, and postoperative BCVA, *P*-values were determined by ANOVA. For MH closure, ELM restoration, and EZ restoration, *P*-value determined by Chi-squared test

For the MHD > 400-μm group, the ELM was restored by 6 months after surgery in 85.7% (6 of 7) in the Hole-Door group, 100% (6 of 6) in the Foveal Flap group, and 33.3% (5 of 15) of the patients in the Negative group (Table 3). Postoperative restoration of the EZ at 6 months occurred in 28.6% (2 of 7) in the Hole-Door group, 33.3% (2 of 6) in the Foveal Flap group, and 13.3% (2 of 15) of the patients in the Negative group. The Negative group had significantly poorer restoration of the ELM than the other two groups (*P* = 0.017, *P* = 0.002), while there was no significant difference for EZ restoration among the three groups (*P* = 0.569). The Hole-Door group and the Foveal Flap group had significantly better final visual acuity than the negative group (*P* = 0.013).

## Discussion

Several authors have recently used iOCT to show changes in MH geometry after ILM peeling [16, 17]. However, the relationship of the intraoperative findings with the MH successful closure rate and anatomic normalization were not thoroughly analyzed. We found three types of iOCT features at the hole edge that were evident after ILM peeling. The morphological characteristics imaged by iOCT are closely related to the prognosis of MH surgery. Thus, the hole-door and foveal flap structures imaged by iOCT during surgery served as positive predictors of MHs that acquired better anatomic and functional results after surgery than did the group in which these features were absent.

Though the nature of the foveal flap is still unknown, it is considered to be an early stage operculum [18, 19]. With the development of posterior vitreous detachment, the foveal flap becomes separated from the retinal tissue as an operculum. Histopathological results suggest that the foveal flap is a part of the retinal tissue [20, 21]. One report stated that good anatomic and functional outcomes were achieved by preserving the foveal flap for the treatment of MH [22]. While preservation of the flap can be achieved by the surgeon using a microscope during the procedure, the use of iOCT may be helpful for making more objective assessments during surgery. We used iOCT to observe the morphology of the foveal flap after ILM removal. Further, iOCT confirmed that all flaps were preserved during the surgery. Subsequent analysis showed that these patients had a better prognosis compared to the negative patients for whom no foveal flap or door-hole was present. We believe that the foveal flap can cover the defect in the inner retina and facilitate hole closure. This then results in a better restoration of photoreceptors at the fovea. To the best of our knowledge, this study is the first to use iOCT to describe the feature of foveal flaps after ILM peeling and demonstrate the beneficial effect on the prognosis after MH surgery.

iOCT showed that 15 patients had vertical pillars of tissue at the edges of the hole projecting into the vitreous cavity after ILM peeling, which is typical of the hole-door MH and predicts postoperative Type 1 closure [23]. In our study, we found that patients with this phenomenon had a better recovery of the foveal microstructure and a better visual outcome compared with the negative group, even though there was no difference in the successful closure rate. Kumar and Yadav [23] considered that the tissue pillars could be composed of redundant retinal tissue, subclinical epiretinal membranes, or small residual pieces of the ILM attached to the edges of the hole. They suggested that the mechanism of closure could be similar to the inverted ILM flap surgical approach in which the pillars provide mechanical support to bridge the gap and more quickly cover up the defects in the inner retina [24–27].

Visual recovery after MH closure may depend on the recovery of the retinal microstructure in the fovea, particularly the outer retina [5–8]. The authors reported that the restoration of the ELM and the EZ lines over the closed MH was associated with better BCVAs. However the presence of hyperreflective bridging tissue at the closed MH indicated that it was closed with scar tissue or glial tissue, including collagen components derived from Müller cells, that migrated in.

The restoration of the ELM in the Hole-Door and Foveal Flap groups was higher than in the Negative group. Correspondingly, both groups had better postoperative visual acuity than the Negative group. Our results show that the favorable visual outcomes after MH surgery were related to restoration of the ELM, and this is similar to previous reports [5–8]. This observation indicated that the recovery of the ELM might be the most critical component for visual function improvement in the early stage after MH surgery.

The size of the hole was also closely related to the prognosis of MH surgery [28]. Regarding preoperative MH size, Liu et al. reported that simply dividing patients into those with MHs > 400 μm and those with MHs ≤400 μm was more clinically significant for the prognosis after MH surgery [8]. Our study shows that the intraoperative feature at the hole edge may be the best predictor of prognosis for those with a MHD > 400 μm. For those in which the MHD was ≤400 μm, there were no significant differences between the ELM restoration, EZ restoration, and BCVA of the three groups.

Real time iOCT is a newly developed technology in the field of ophthalmic imaging. The 3-year results of the DISCOVER study found that 69% of posterior segment surgeons preferred viewing static images on the display screen, and that percent increased from year 1 to subsequent years. These may be related to greater OCT detail and subtle changes on screen review than in real time. Therefore, non-real time iOCT continues to have important clinical value in MH surgery.

This study has the following limitations. It was a retrospective study and involved a small number of cases, which limited the statistical strength of the analysis. In addition, the imaging system used in this study was not integrated into the microscope, which may have impacted the overall functionality of iOCT in these cases.

## Conclusions

We used iOCT as a novel approach to image the edge of MHs after ILM peeling during surgery. This approach can enable the surgeon to identify MH edges composed of hole-door, foveal flap, or neither configuration, and it can provide useful predictive information for postoperative restoration of the retinal microstructure and visual outcomes of MHs, especially large ones. Our results also provides useful insights into the pathophysiology of ILM peeling in MH surgery.

## Data Availability

The datasets for the analysis of the current study are readily available from the corresponding author on reasonable request.

## Abbreviations

iOCT: Intraoperative Optical Coherence Tomography
MH: Macular Hole
EZ: Ellipsoid Zone
ELM: External Limiting Membrane
BCVA: Best Corrected Visual Acuity
